# Investigating Health and Well-Being Challenges Faced by an Aging Workforce in the Construction and Nursing Industries: Computational Linguistic Analysis of Twitter Data

**DOI:** 10.2196/49450

**Published:** 2024-06-05

**Authors:** Weicong Li, Liyaning Maggie Tang, Jed Montayre, Celia B Harris, Sancia West, Mark Antoniou

**Affiliations:** 1 The MARCS Institute for Brain, Behaviour and Development Western Sydney University Penrith Australia; 2 School of Architecture and Built Environment The University of Newcastle Callaghan Australia; 3 Centre of Evidence-based Practice for Health Care Policy The Hong Kong Polytechnic University Hung Hom China (Hong Kong); 4 School of Nursing and Midwifery Western Sydney University Penrith Australia; 5 Centre for Work Health and Safety New South Wales Government Gosford Australia

**Keywords:** social media, construction, nursing, aging, health and well-being, Twitter

## Abstract

**Background:**

Construction and nursing are critical industries. Although both careers involve physically and mentally demanding work, the risks to workers during the COVID-19 pandemic are not well understood. Nurses (both younger and older) are more likely to experience the ill effects of burnout and stress than construction workers, likely due to accelerated work demands and increased pressure on nurses during the COVID-19 pandemic. In this study, we analyzed a large social media data set using advanced natural language processing techniques to explore indicators of the mental status of workers across both industries before and during the COVID-19 pandemic.

**Objective:**

This social media analysis aims to fill a knowledge gap by comparing the tweets of younger and older construction workers and nurses to obtain insights into any potential risks to their mental health due to work health and safety issues.

**Methods:**

We analyzed 1,505,638 tweets published on Twitter (subsequently rebranded as X) by younger and older (aged <45 vs >45 years) construction workers and nurses. The study period spanned 54 months, from January 2018 to June 2022, which equates to approximately 27 months before and 27 months after the World Health Organization declared COVID-19 a global pandemic on March 11, 2020. The tweets were analyzed using big data analytics and computational linguistic analyses.

**Results:**

Text analyses revealed that nurses made greater use of hashtags and keywords (both monograms and bigrams) associated with burnout, health issues, and mental health compared to construction workers. The COVID-19 pandemic had a pronounced effect on nurses’ tweets, and this was especially noticeable in younger nurses. Tweets about health and well-being contained more first-person singular pronouns and affect words, and health-related tweets contained more affect words. Sentiment analyses revealed that, overall, nurses had a higher proportion of positive sentiment in their tweets than construction workers. However, this changed markedly during the COVID-19 pandemic. Since early 2020, sentiment switched, and negative sentiment dominated the tweets of nurses. No such crossover was observed in the tweets of construction workers.

**Conclusions:**

The social media analysis revealed that younger nurses had language use patterns consistent with someone experiencing the ill effects of burnout and stress. Older construction workers had more negative sentiments than younger workers, who were more focused on communicating about social and recreational activities rather than work matters. More broadly, these findings demonstrate the utility of large data sets enabled by social media to understand the well-being of target populations, especially during times of rapid societal change.

## Introduction

### Background

Construction and nursing are 2 critical industries for the Australian economy and worldwide. Healthy construction workers are needed to build homes, commercial stores, and public infrastructure, affecting transport, health care services, commerce, recreation, and all aspects of daily life. A healthy and functioning nursing workforce is an essential resource for public health, as recognized by the World Health Organization (WHO). In the recent *2022 Skills Priority List* [1], which identified the most in-demand professions of the coming decade, both professions were listed in the top 10; specifically, construction managers claimed the top spot, whereas registered nurses secured the fourth position. Although both industries undeniably serve critical functions, there are immediately apparent differences between the 2 industries. These include the nature of the work, size of the organizations (60% of construction businesses are sole traders and 98.6% have <20 employees), and demographics of the 2 workforces (most construction workers are male, and most nurses are female) [2]. Despite these differences, the 2 industries face common challenges. They both have aging workforces, face worldwide labor shortages, involve work that is physically demanding and stressful, and need to retain and transfer older workers’ expert knowledge. Prior work suggests that workers across these 2 industries face different work-related pressures and stress (particularly during the COVID-19 pandemic), which in turn are likely to impact their job performance, health, and career decisions [3]. The goal of this paper is to empirically examine social media use in the construction and nursing industries to provide insights into the challenges workers are facing and to use big data analytics to compare issues between industries, across age groups, and within Australia versus overseas.

### Research Using Text Data From Social Media Platforms

Social media platforms provide workers with channels to express their experiences and feelings [4]. Social media networks may provide insights into the state of mind and the experiences of users. Such insights are typically obtained by conducting surveys or interviews with the samples drawn from the population or populations of interest. Although social media data analytics do not replace traditional face-to-face data collection methods such as surveys or interviews, they complement such approaches and make it possible to explore issues in ways that are not possible using more traditional approaches. First, because social media platforms are digitized, large data sets can be obtained for empirical research. Therefore, trawling social media data has become one of the best ways to analyze and predict trends within industries [5]. Second, social media makes it possible to compare large data sets both across time and large geographic distances. Third, the data can be subjected to state-of-the-art computational linguistic and natural language processing techniques to understand the pressures and risks that workers face within a given industry at a particular time and whether these challenges differ for subgroups within an industry (eg, younger vs older workers). The knowledge gained from such analyses can inform the design of interventions and policies to safeguard workers from potential harm. For these reasons, social media data are being used to explore issues related to work pressure, performance, career advancement, and work health and safety.

Traditionally, the construction industry has not been closely associated with the use of social media platforms, but construction companies are increasingly using social media to improve visibility and build brand awareness [6], share safety knowledge [7], enhance daily operations [8], measure students’ attitudes [9], and gauge public opinion of projects. Although natural language processing is a growing area within the construction industry [10], only a few studies have applied computational linguistic techniques to social media data sets [5,11]. A recent systematic review of the literature on big data studies within construction called for research on how social media big data analytics can be used to prevent threats such as safety issues, injury, or mental illness caused by work-related stress [12]. This study directly addresses this need.

In nursing, numerous studies have examined the use of social media by nurses for a variety of purposes [13], including delivering health care to patients [14,15], training nursing students [16], and dissemination of communications during conferences [17]. However, the possibility of analyzing nurses’ activity on social media platforms such as Twitter (subsequently rebranded as X) to understand their experiences of workplace stress and risks to their work health and safety has received surprisingly little attention to date.

An analysis of 53,063 tweets from January 2019 to December 2020 revealed that nurses experienced more frequent and intense negative emotions (eg, decreased joy and increased sadness, fear, and disgust) than in the year preceding the COVID-19 pandemic [18]. Notably, fear preceded the COVID-19 pandemic waves by 2 weeks, suggesting that frontline workers are finely attuned to increases in work pressure even in an already high-stress work environment. This has implications for preventing fatigue, burnout, and mental health disorders related to unhealthy or unsustainable work conditions via sensitive detection of changes to well-being at the population level.

An analysis of 4.5 million tweets posted by US and UK nurses revealed that health care providers in the 2 countries experienced common challenges concerning public health (eg, policy and COVID-19–related pressures), social values (related to aspects of health), and political issues related to the COVID-19 pandemic (more positive in the United Kingdom) [19]. However, the experiences in the 2 countries reflected local sociopolitical trends and the cultural norms regarding emotional display (more accepted in the United States and more reserved in the United Kingdom). Both countries showed sharp increases in fear and sadness during the first wave of the COVID-19 pandemic and when there was an increase in the virus reproduction rates. Fear gradually reduced with time, but sadness was maintained. Anger was experienced by both groups in response to a rise in the number of COVID-19 deaths.

This raises the possibility that nurses are particularly vulnerable to poor mental health (eg, concerns, fear, and anxiety) due to the nature of their work, characterized by long working hours, burnout, loneliness, fatigue, and occupational stigma [20,21]. These risks are magnified when needing to manage a long-lasting emergency situation such as the COVID-19 pandemic, often with a lack of resources and under more demanding conditions [22]. While nurses have been praised for their frontline efforts in the care and treatment of patients with COVID-19, very little is known regarding how nurses have responded to the current emergency and how this is reflected in the language of their social media posts [23]. Furthermore, nurses have not been compared to workers in other industries. This study will directly address this knowledge gap.

### Comparing Age Groups and Industries

Studies comparing work health and safety issues across different subgroups of workers (eg, younger vs older workers) and industries with diverse demographical profiles (eg, nursing and construction) are scarce. To our knowledge, this is the first study to compare construction and nursing industries with the goal of developing a detailed understanding of both the common and unique challenges faced by workers in these critical industries and how these challenges and risks interact with aging.

Age may be a key differentiator between workers that impacts their mental health and response to stress. In general, among health care workers [24], older adults compared to younger adults show better mental health and emotion regulation [25], including during the COVID-19 pandemic [26]. This suggests that although older workers are frequently depicted as frail and vulnerable, broader evidence from aging research suggests they could be more resilient and better able to adapt and accommodate periods of instability than younger workers. In prior work, we conducted a scoping literature review to understand the physical and mental factors that affect older construction workers’ work ability [2]. We found that the literature was dominated by studies focusing on physical health factors (eg, hearing loss, muscle pain, respiratory issues, and conditions resulting from prolonged work in and around construction sites), and although there was evidence of mental health risks and harms [27], these were less well understood, including their interaction with physically debilitating conditions (eg, diminished mental health due to work-related stress is a precursor of physical injuries and chronic pain).

In contrast to the paucity of research on the mental health of construction workers, many reviews have been published on the work health and safety of nurses, including reviews focusing specifically on older nurses [28,29]. However, most of these reviews focused on macrolevel issues that affect workers at the organizational level. Consequently, in a systematic review of evaluated programs and interventions intended to support the health, well-being, and retention of aging nurses, we discovered that the interventions were often mismatched to the needs of the nurses the program was trying to address [30]. For example, wellness interventions that focus on healthy eating or yoga are unlikely to address the underlying systemic workplace challenges that nurses encounter as they age at work. Furthermore, support programs and interventions for nurses need to be both sustainable at the organizational level as well as adaptable to the workers’ changing circumstances, as determined by their health and aging.

In addition, a recent survey of construction workers and nurses within New South Wales revealed that nurses were far more likely to experience the effects of stress, burnout, and workload pressures (LM Tang et al, unpublished data, September 2021). These effects were exacerbated by the COVID-19 pandemic in nurses, whereas construction workers reported being far less affected by the pandemic. Age differences were also observed.

Taken together, the findings from the abovementioned studies provide converging evidence from which a picture is beginning to emerge concerning the pressures under which workers in each industry are having to operate.

### This Study

In this study, we conducted a detailed linguistic analysis of a large data set of social media posts from younger and older individuals working in the construction and nursing industries. Industry membership was determined by sourcing data from industry-based groups and Twitter hashtags and then filtering the data. Big data analytics and computational linguistics were used to examine patterns of word use relating to industry, age, location, and mental and physical health. Content and sentiment analyses allowed us to determine common issues discussed on social media across individuals working in nursing and construction industries and how they are associated with age. On the basis of the emerging picture of the work pressures within the nursing and construction industries reviewed in the earlier sections, we hypothesized that our analysis of tweets would detect greater effects of stress, burnout, and workload pressures in nurses, and these mental health effects would be exacerbated by the COVID-19 pandemic. An additional consideration was whether any observed barriers to well-being would vary across ages (ie, younger vs older workers) and locations (ie, Australia vs overseas).

## Methods

### Ethical Considerations

This study was conducted in full compliance with the National Statement on Ethical Conduct in Human Research and approved by the Western Sydney University Human Ethics Committee (approval number: H14518).

### Platform

**Figure 1:**
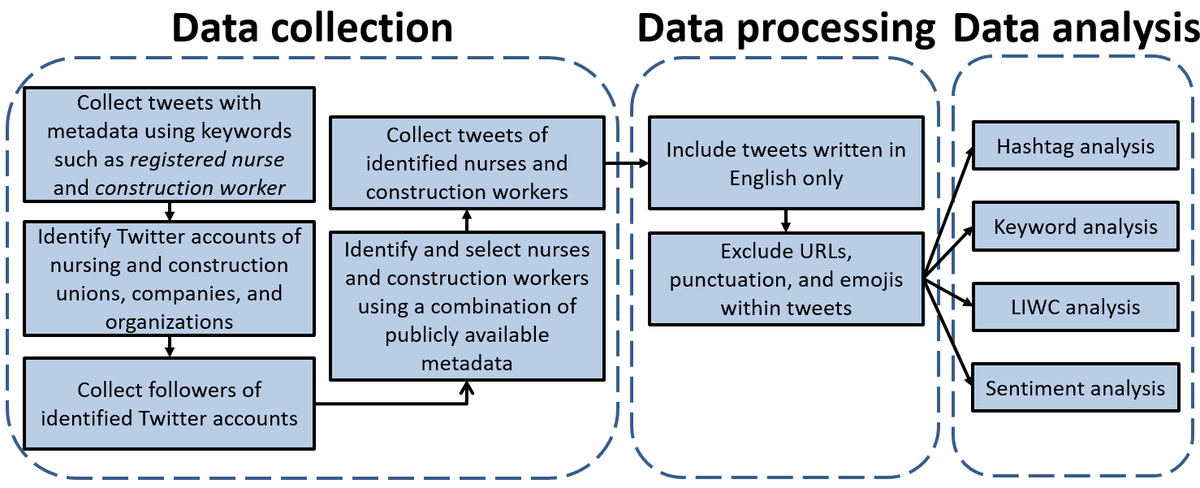
Data collection, processing, and analysis workflow. LIWC: Linguistic Inquiry and Word Count.

Twitter is a social media platform that allows users to publish short posts, called tweets, consisting of up to 280 characters about any topic, including their thoughts, daily activities, political opinions, and news. Twitter enables users to follow others without requiring confirmation, making tweets public. In total, >240 million active users access the Twitter service. Approximately 6000 tweets are posted every second, which equates to 350,000 tweets per minute, 500 million tweets per day, and a staggering 200 billion tweets per year [31]. It has become one of the most important social media platforms in the world. At the time, Twitter had a publicly available application programming interface that developers and researchers could access to download Twitter data that fit a specific combination of criteria. For this study, data collection and processing were performed as illustrated in Figure 1. Notably, there were 2 steps: first, the data were harvested and filtered, and second, we performed data analytics, processing, and mining knowledge from the data.

### Population

To identify workers within construction and nursing professions, we first collected tweets together with their metadata using keywords such as *registered nurse* and *construction worker*. From these tweets and metadata, we identified a list of Twitter accounts of the relevant unions, companies, and organizations for each industry. Then, we collected the large lists of followers of these accounts. Next, we identified which of these followers were construction workers and nurses and used a combination of their publicly available metadata to determine their location (in or outside Australia) and their age category (eg, using their name and short biographical profile). This process was partially automated but also involved a manual component, particularly for determining the age category (>45 years or <45 years). We adopted the definition provided by the Australian Department of Employment and Workplace Relations [32], which categorizes mature-age workers as those aged >45 years. Determining the age of users involved some combination of looking at the user’s profile picture, name or handle, cross-referencing with other social media accounts (eg, LinkedIn [Microsoft Corp] or Facebook [Meta Platforms, Inc]) or professional profile pages (eg, company, hospital, and university websites) that provide age data, or some proxy for estimating age (eg, the year a professional qualification was awarded). Users for whom we could not confidently determine their age category were excluded.

### Data Collection

Data were collected from the Twitter platform using the Twitter application programming interface and were subject to the privacy policy regarding the release of the data held by the social media platforms to the public. Furthermore, keywords such as *construction worker* and *registered nurse* were used to retrieve potential nurses and construction workers and their tweets as an initial step. Metadata including username; name; user ID; language; hashtags; tweet time; and number of likes, replies, and retweets were collected simultaneously using *Twint* and *Tweepy*, Twitter scraping packages written in Python (Python Software Foundation) that allow for data collection from Twitter profiles.

### Data Filtering and Preparation

Only tweets written in English were included in the analyses. URLs, punctuation marks, symbols, and emojis were removed. *Stanza* developed by Stanford Natural Language Processing Group and Python *Natural Language Toolkit* package were used for tokenization (ie, breaking a sentence into small units for subsequent analysis), removing stop words (eg, are, is, an, this, and that; the complete list is available on GitHub [33]), sentiment analysis, and monogram and bigram keyword analysis (ie, 1 word vs 2 words that co-occur, eg, bigrams involving mask included “wear mask” vs “anti mask”).

### Analysis

#### Hashtag Topics

A hashtag is a word or combination of words preceded by a # symbol, which is used to index topics on Twitter (eg, #construction, #nursing, and #COVID). This function was created on Twitter and allows people to easily follow topics they are interested in. We took advantage of this functionality to conduct a series of data visualizations and quantitative analyses to explore trending topics within and across industries and age categories.

#### Keywords

In addition to the abovementioned hashtag analysis, we analyzed the keywords contained in tweets (ie, the content words that remained after stop words, punctuation marks, and so on were removed). This analysis provided a more detailed exploration of the themes raised by the hashtag topic analyses.

#### Word Counts Analysis

The Linguistic Inquiry and Word Count (LIWC) application [34] is the most widely used corpus of dictionaries for computational linguistic analyses of text data. Numerous studies on mental health topics using LIWC have shown strong evidence of particular patterns of language use that are highly relevant to certain mental health issues. Notably, LIWC compares words appearing in an input text file with the words listed in its customizable dictionary. Then, LIWC uses its algorithms to sort words into predefined and psychologically meaningful categories and performs a series of calculations. The end result is a summary of the statistical distribution of words within a given text into those that fall into LIWC categories, which include function words, pronouns, impersonal pronouns, verbs, auxiliary verbs, and past-tense words.

For this study, we used the latest version of LIWC (LIWC-22; Pennebaker Conglomerates, Inc) and the built-in English dictionary. Tweets containing <10 words were not included in the LIWC analysis, as they are unlikely to satisfy the requirements of LIWC and will not produce meaningful results.

#### Sentiment Analysis

Sentiment analysis was conducted using *Stanza*, the Python package of Stanford Core Natural Language Processing Group [35,36], which is based on deep learning and has been shown to have state-of-the-art performance [37]. Sentiment analysis is a powerful technique used to understand the public opinion of social media users and is suitable for use with short text posts such as tweets [38]. Using this approach, text can be classified as either positive, negative, or neutral sentiments. Examples of positive, negative, and neutral sentiments are as follows:

Great choice and important area that needs exploring. I would be confident even if you found someone exploring similar, you would be able to provide unique new knowledge. The anxiety element is real and recognised though! Good luck and feel free to DM.

Positive sentiment

Nope, so much bad energy used to devalue and treat ordinary Australians like cattle. Fascism, led by the religious right, will destroy this country. There hasn’t been a time, in my life, when AU wellbeing is so low.

Negative sentiment

I was watching a show on tv last night re improving mental health which was timely given the day I had yesterday. It mentioned effects of social media on mental health.

Neutral sentiment

## Results

### Data Summary

The data set consisted of 1,505,638 tweets from 395 Twitter users, as presented in Multimedia Appendix 1. The data were collected across a period spanning 54 months, from January 2018 to June 2022, which equates to approximately 27 months before and 27 months after the WHO declared COVID-19 a global pandemic on March 11, 2020. Data were separated into those that originated in Australia versus those that originated in other parts of the world because tweets are known to reflect local sociopolitical trends and cultural norms (eg, regarding the display of emotions [19]). Furthermore, we divided users into younger and older workers to explore whether the challenges that workers face interact with age.

### Hashtag Topics

The large data set contained a wide variety of hashtag topics. Overall, younger workers tended to use a wider variety of hashtag topics than older workers, and this pattern held for both industries. Comparing industries, nurses tended to use more hashtag topics than construction workers (Multimedia Appendix 2). Our large data set contained thousands of hashtag topics, with a very long tail in their distribution (ie, some obscure hashtags had only 1 or 2 times). To reduce the data for analysis, we grouped the top 200 hashtag topics into groups based on themes, and 10 unique thematic categories emerged (Multimedia Appendix 3).

Political hashtags were popular in both industries. In particular, older workers made greater use of political hashtags than younger workers, and this difference was especially pronounced in construction workers. Younger construction workers were by far the most likely to tweet about “life,” including various entertainment activities, such as sports, eating out, camping, television shows, shopping, hobbies, and pets. This suggests the presence of a work-life balance disparity across the 2 industries. Delving deeper, we found that construction workers had a work-life topics ratio >1, whereas the ratio for nurses was <1, indicating that work was taking a disproportionate amount of nurses’ time and attention, and this could be interpreted as an indicator of nurses being overworked. This pattern was exacerbated in the younger groups of workers: the work and life topics ratio of younger construction workers was 5 times higher than that of younger nurses.

Consistent with the expectations, nurses used COVID-19–related hashtags (eg, #COVID-19, #coronavirus, #mask, #vaccination, #lockdown, and #ppe) to a far greater extent than construction workers. This likely reflects their practice scope, expertise, and daily experiences in the course of performing their professional duties. Furthermore, nurses were more likely to include hashtags in their tweets falling within the “campaign” and “organization” categories, which included social media calls for action to improve working conditions (not relating to political purposes) and to voice support. Nurses, and especially older nurses, were more likely to use “positive words” in their hashtags, which consisted of SMS text messages voicing support for colleagues and others facing challenging circumstances in their industry (eg, #kind, #kindnessmatters, #respect, #grateful, #empathy, and #teamwork).

As shown in Table 1, nurses were far more likely to tweet hashtags about their well-being; mental health issues; and the deleterious effects of burnout, fatigue, and tiredness. Younger nurses tended to focus on mental health issues and burnout or fatigue, whereas older nurses were more likely to tweet about well-being. Older construction workers were more likely to tweet about pains, aches, and physical health issues.

**Table 1:** Hashtag topics used by younger and older nurses and construction workers directly related to health, well-being, and the COVID-19 pandemic (n=349,790).

Hashtag topic	Nurses (n=217,510, 62.18%), n (%)			Construction workers (n=132,460, 37.82%), n (%)	
	Younger (n=152,570, 70.14%)	Older (n=64,940, 29.86%)	Younger (n=77,929 58.83%)		Older (n=54,531, 41.67%)
Burnout, fatigue, tiredness, and sleep issues	93 (0.06)	37 (0.06)	10 (0.01)		6 (0.01)
Pain, ache, and physical health	42 (0.03)	10 (0.01)	10 (0.03)		52 (0.10)
Mental health	479 (0.31)	100 (0.15)	26 (0.03)		14 (0.03)
Well-being	210 (0.14)	189 (0.29)	17 (0.02)		2 (0.004)
COVID-19 pandemic	7031 (4.61)	1723 (2.65)	1488 (1.91)		1132 (2.08)

Tweets with hashtag topics had a higher average number of likes, replies, and retweets than topics without hashtags (Multimedia Appendix 4). Furthermore, tweets containing hashtag topics related to health and well-being had a higher average number of likes and retweets than those with hashtag topics other than health and well-being. Overall, tweets with hashtag topics received more attention and engagement, especially those about health and well-being topics.

### Keywords

#### Monogram Keywords

Monogram keywords refer to individual content words within tweets that convey meaning. Compared to hashtags, keywords are more indicative of the content of the tweet, in that they better reflect the author’s intended meaning. A summary of the monogram keyword data is provided in Multimedia Appendix 5.

Consistent with what was observed for hashtags, monogram keyword counts revealed that nurses made greater use of keywords associated with burnout, health issues, and mental health compared to construction workers (Table 2). Compounding the above, COVID-19 also had a huge effect on nurses, and this was especially pronounced for younger nurses.

**Table 2:** Monogram keywords used by younger and older nurses and construction workers directly related to health, well-being, and the COVID-19 pandemic (n=9,076,641).

Monogram keywords	Nurses (n=5,621,926, 61.94%), n (%)			Construction workers (n=3,454,715, 38.06%), n (%)	
	Younger (n=3,979,076, 70.78%)	Older (n=1,642,850, 29.22%)	Younger (n=2,167,081, 62.73%)		Older (n=1,287,634, 37.27%)
Burnout, fatigue, tiredness, and sleep issues	6066 (0.15)	1466 (0.09)	1643 (0.08)		724 (0.06)
Pain, ache, and physical health	6989 (0.18)	2410 (0.15)	1063 (0.05)		670 (0.05)
Mental health	7113 (0.18)	2256 (0.14)	1682 (0.08)		791 (0.06)
Well-being	622 (0.02)	802 (0.05)	112 (0.005)		57 (0.004)
COVID-19 pandemic	34,084 (0.86)	13,516 (0.82)	4484 (0.21)		4132 (0.32)

An analysis of likes, replies, and retweets revealed that tweets containing health and well-being keywords were more likely to gain the attention of other Twitter users, resulting in more engagement (Multimedia Appendix 6).

Monogram keywords directly related to physical and mental health and well-being were visualized as word clouds to illustrate the differences between industries and the age subgroups (Multimedia Appendix 7). Within each word cloud, the size of each word represents its frequency of occurrence, and the density of the cloud depicts the number of different keywords used. Differing patterns were observed for the 4 subgroups. Younger workers in both industries had word clouds that were denser, indicating that they posted about a wider variety of topics and problems. Younger nurses tweeted most frequently about “mental” and “sleep,” whereas older nurses mentioned “pain” and “well-being” to a far greater extent and placed much less focus on “sleep.” Among construction workers, both younger and older workers mentioned “sleep,” “pain,” and “tired.” The older construction workers had the sparsest word cloud.

#### Bigram Keywords

Bigram keywords refer to pairs of content words that co-occur within tweets and convey additional context and the author’s intended meaning (eg, the monogram “mask” has different connotations in the bigrams “wear mask” vs “anti mask”). A summary of bigrams is presented in Multimedia Appendix 8. Nurses consistently used more bigrams than construction workers.

Consistent with what was observed for hashtag topics and monograms, nurses made greater use of bigram keywords associated with burnout and physical and mental health issues (Table 3). Furthermore, the COVID-19 pandemic disproportionately affected nurses, and this effect was magnified for younger nurses.

**Table 3:** Bigram keywords used by younger and older nurses and construction workers directly related to health, well-being, and the COVID-19 pandemic (n=3,528,198).

Monogram keywords	Nurses (n=2,155,270, 61.09%), n (%)			Construction workers (n=1,372,928, 38.91%), n (%)	
	Younger (n=1,490,264, 69.15%)	Older (n=665,006, 30.85%)	Younger (n=891,042, 64.9%)		Older (n=481,886, 35.1%)

Burnout, fatigue, tiredness, and sleep issues	2093 (0.14)	551 (0.08)	614 (0.07)		315 (0.06)
Pain, ache, and physical health	3371 (0.23)	1038 (0.16)	591 (0.07)		236 (0.05)
Mental health	4882 (0.33)	1510 (0.23)	1023 (0.11)		564 (0.12)
Well-being	498 (0.03)	447 (0.07)	49 (0.005)		24 (0.005)
COVID-19 pandemic	12,281 (0.82)	4036 (0.61)	1571 (0.18)		1520 (0.32)

Bigram word clouds related to physical and mental health issues and well-being are presented in Multimedia Appendix 9. The bigrams reveal additional details that were obscured in the monogram clouds. “Mental health” was the most frequently occurring bigram for all groups. “Mental illness” featured prominently for younger nurses and both groups of construction workers, but “mental well-being*”* occurred more frequently for older nurses. Furthermore, older nurses posted about physical well-being more than the other groups. Notably, density increased for the older groups relative to their monogram clouds. Younger nurses still had the densest cloud, and the bigrams within indicated that they were experiencing the most distress of all the groups (eg, “fall asleep,” “I am tired,” “not sleep,” “sleep tonight,” and “pain relief”). Older construction workers made frequent reference to specific medical conditions such as cardiovascular disease. Younger construction workers posted about nerve pain and neuropathic pain, likely related to the physical nature of construction work.

### LIWC Results

For the LIWC analyses, tweets were grouped into those that did or did not contain health and well-being keywords. In Multimedia Appendix 10, we present the basic LIWC summary variables. Authenticity scores suggest that tweets were equally truthful regardless of whether the tweet was about a health-related topic or other topics. Analytical thinking, clout, and positive emotional tone were greater in tweets about non–health-related topics.

As presented in Multimedia Appendix 11, overall, tweet authors used more first-person singular pronouns and affect words when posting about health and well-being. Increased use of first-person singular pronouns is a reliable predictor of elevated levels of stress, depression, and suicidal ideation [39,40]. Furthermore, we found more affect words in health-related tweets. As expected, non–health-related tweets contained more words associated with the social category.

LIWC analyses of positive versus negative emotions and tone revealed that tweets about health and well-being were far more likely to convey negative emotions, perhaps reflecting fear, despair, sadness, or anger (Multimedia Appendix 12). Central to our research question and aims, we observed that words falling within the LIWC categories of *health*, *fatigue*, *illness*, *wellness*, and *mental* were far more likely to occur in tweets about health and well-being.

Then, we further examined the LIWC indicators for the tweets with health and well-being keywords. Comparing younger and older workers across industries, we observed that younger nurses showed language use patterns, suggesting that they were experiencing the deleterious effects of suboptimal work conditions (Multimedia Appendix 13). Younger nurses used words within the *fatigue*, *illness*, and *mental* categories more often than their older colleagues. Older nurses made greater use of words in the *wellness* category. These differences all reached statistical significance (Table 4). In contrast, no differences were found between the younger and older construction workers. In general, construction workers spent less time posting about health and well-being topics, and no differences were observed between younger and older construction workers.

**Table 4:** ANOVA tests for the Linguistic Inquiry and Word Count (LIWC) indicators health, fatigue, illness, wellness, and mental comparing younger versus older workers in the nursing and construction industries.

LIWC indicators	Younger vs older nurses		Younger vs older construction workers	
	*F* test (*df*)	*P* value	*F* test (*df*)	*P* value
Health	1.18 (1, 16,183)	.28	2.72 (1, 4138)	.10
Fatigue	21.82 (1, 16,183)	<.001	0.33 (1, 4138)	.56
Illness	17.12 (1, 16,183)	<.001	0.52 (1, 4138)	.47
Wellness	228 (1, 16,183)	<.001	0.07 (1, 4138)	.79
Mental	18.58 (1, 16,183)	<.001	0.08 (1, 4138)	.78

### Sentiment Analysis

**Figure 2:**
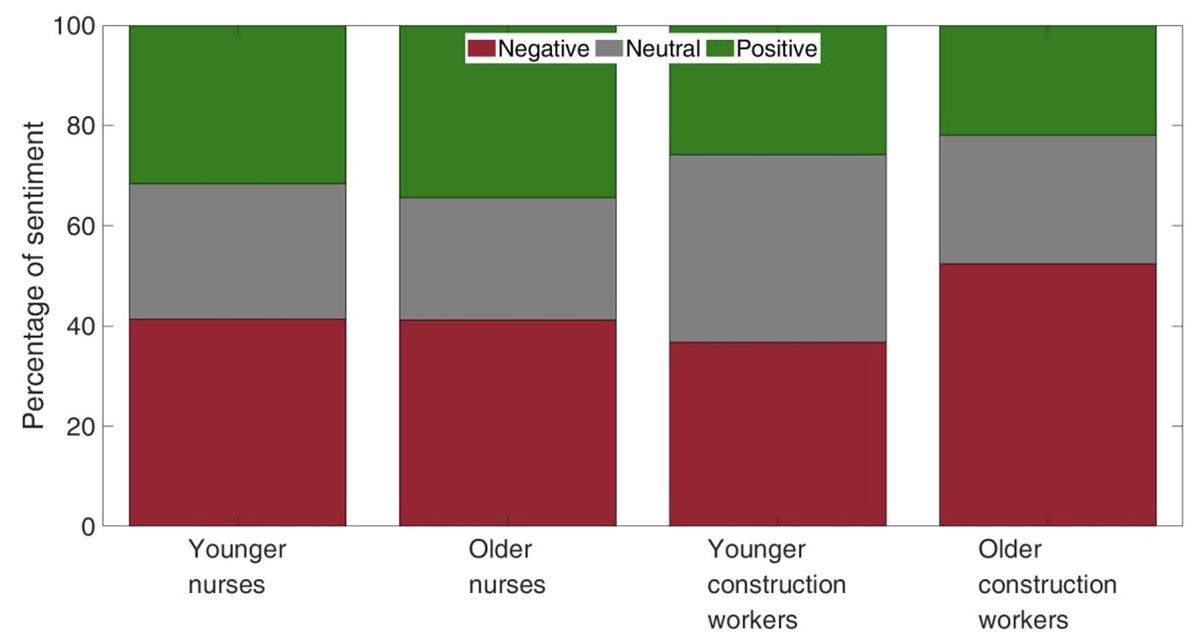
Percentage of tweets with negative, neutral, and positive sentiments among younger and older nurses and construction workers.

Sentiment analysis revealed that, overall, older construction workers made more tweets with negative sentiment (Figure 2). Younger construction workers were the most neutral. Older nurses tended to be more positive. Other than that, the differences between the younger and older nurses were relatively minor.

Next, we conducted a sentiment analysis of the tweets that did or did not contain keywords related to health and well-being. The right panel of Multimedia Appendix 14 presents the tweets without health and well-being keywords and serves as a baseline. The left panel of Multimedia Appendix 14 shows an increase in negative sentiment when tweeting about health or well-being topics. Younger nurses and older construction workers were the most negative, whereas older nurses were the most positive.

### Australian Workers Versus Non-Australian Workers (LIWC Analysis)

To explore whether our observations were specific to the Australian context or reflected trends observable in other nations, we compared LIWC categories in Australian and non-Australian workers (Multimedia Appendix 15). In total, 51.5% (53/103) of the non-Australian construction workers come from Europe and North America, whereas 48.5% (50/103) come from Africa, Asia, and Latin America. Regarding nurses, 75% (78/104) of the non-Australian workers come from Europe and North America, and 25% (26/104) come from Africa, Asia, and Latin America. We observed that Australian workers in both industries used more words falling within the *mental* category, which was significant as confirmed by ANOVA tests (Table 5).

**Table 5:** ANOVA tests for the Linguistic Inquiry and Word Count (LIWC) indicators health, fatigue, illness, wellness, and mental comparing younger versus older workers in the nursing and construction industries.

LIWC indicators	Australian nurses vs non-Australian nurses					Australian construction workers vs non-Australian construction workers			
	Younger nurses		Older nurses		Younger construction workers			Older construction workers	
	*F* test (*df*)	*P* value	*F* test (*df*)	*P* value	*F* test (*df*)		*P* value	*F* test (*df*)	*P* value
Health	2.69 (1, 12329)	.10	4.79 (1, 3852)	.03	0.1 (1, 2732)		.75	59.95 (1, 1404)	<.001
Fatigue	3.64 (1, 12329)	.06	2.17 (1, 3852)	.14	8.04 (1, 2732)		.005	15.8 (1, 1404)	<.001
Illness	1.42 (1, 12329)	.23	46.05 (1, 3852)	<.001	14.98 (1, 2732)		<.001	22.59 (1, 1404)	<.001
Wellness	0.59 (1, 12329)	.44	64.73 (1, 3852)	<.001	18.59 (1, 2732),		<.001	32.78 (1, 1404)	<.001
Mental	23.85 (1, 12329)	<.001	55.57 (1, 3852)	<.001	30.99 (1, 2732)		<.001	23.44 (1, 1404)	<.001

This could be a sign of greater stress in Australian workers, potentially leading to future mental health concerns. Furthermore, Australians made more frequent reference to *health* and *illness* (with the exception of younger construction workers), suggesting that their health had been adversely affected, or at least that it was on their mind. Use of *health-*, *fatigue-*, *illness-,* and *wellness*-related words by younger nurses within and outside Australia were similar, whereas older Australian nurses made significantly more use of *health-* and *illness-related* words and less use of *wellness-related* words than their peers outside Australia. Similar results were found for older Australian construction workers compared to those outside Australia (except for more use of wellness-related words). On the other hand, younger Australian construction workers had less use of *fatigue-* and *illness-related* words, which might suggest that they are in a better condition compared to their colleagues worldwide.

### The COVID-19 Pandemic Influence

**Figure 3:**
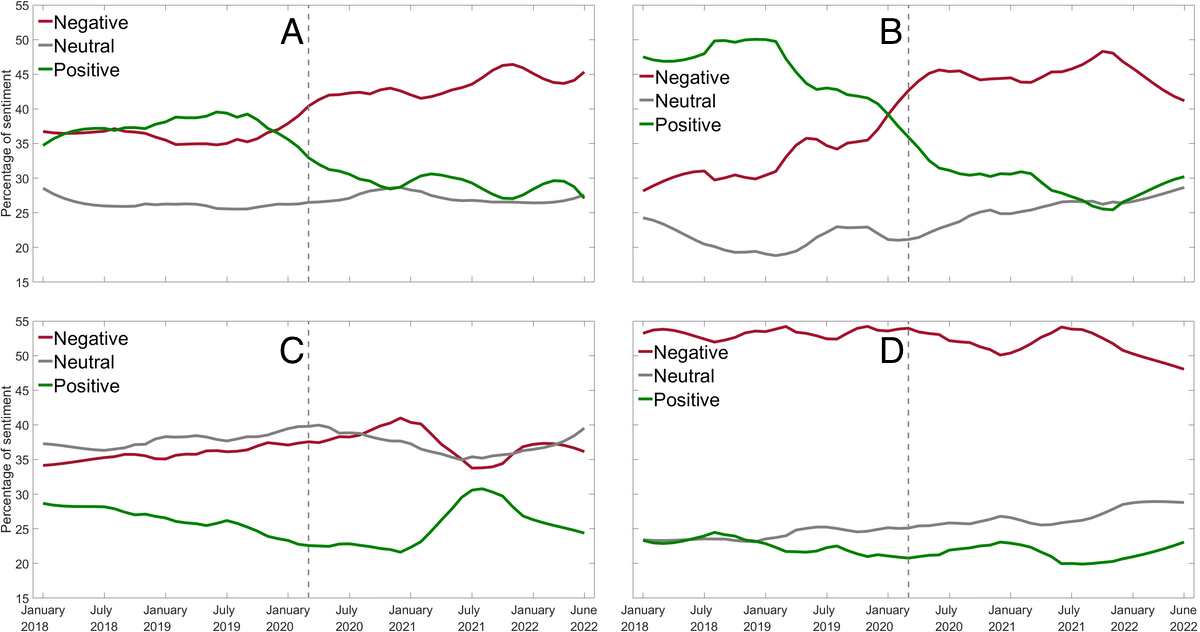
Percentage of tweets with negative, neutral, and positive sentiment changes by month for (A) younger nurses, (B) older nurses, (C) younger construction workers, and (D) older construction workers. The dashed vertical line indicates the announcement of the COVID-19 pandemic by the World Health Organization on March 11, 2020.

The average number of tweets per user over the 54-month period between January 2018 and June 2022 increased more for both younger and older nurses than construction workers, especially since the COVID-19 pandemic. Furthermore, we observed marked differences in the 4 subgroups regarding sentiment at baseline and changes that occurred during the COVID-19 pandemic (Figure 3). Overall, nurses had a higher proportion of positive sentiment in their tweets than construction workers. However, this changed markedly in early 2020 as the positive and negative sentiments crossed over in the months leading up to the WHO’s declaration of COVID-19 as a global pandemic. Since that time, negative sentiment dominated the tweets of nurses. No such crossover was observed among construction workers, in part because both younger and older construction workers consistently had more negative sentiments dating back to January 2018.

**Figure 4:**
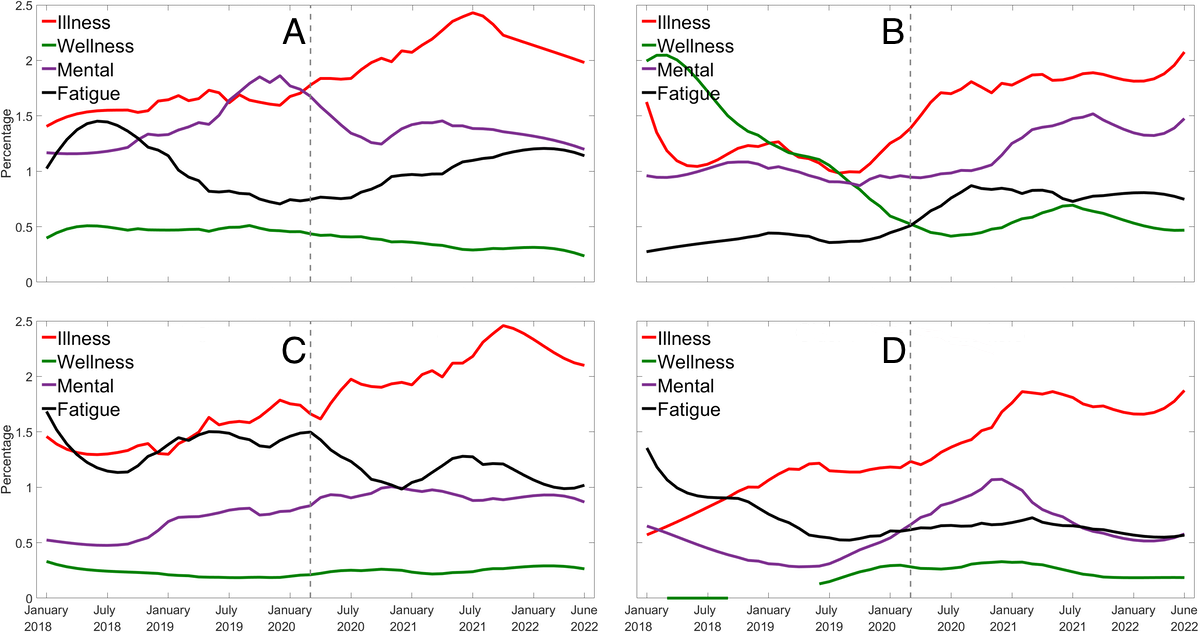
Changes in the Linguistic Inquiry and Word Count (LIWC) indicators illness, wellness, mental, and fatigue by month for (A) younger nurses, (B) older nurses, (C) younger construction workers, and (D) older construction workers. The dashed vertical line indicates the announcement of the COVID-19 pandemic. The calculation of LIWC indicators used only the tweets containing health and well-being keywords.

For all groups, LIWC for *illness*, *mental,* and *fatigue* categories were the most prominent, whereas the LIWC for the *wellness* category either declined or remained low across the 54-month period (Figure 4). For the older nurses, LIWC for the *wellness* category decreased but showed a bump that peaked in mid-2021, although the peak was considerably lower than the pre–COVID-19 pandemic levels. Younger nurses showed a substantial increase in *mental* words leading up to the WHO’s COVID-19 pandemic declaration in March 2020. Younger construction workers were the only group for whom the *fatigue* category had a higher percentage than the *mental* category, indicating that their focus was on their physical health rather than mental health during the sampling period.

## Discussion

### Principal Findings

This is the first social media analysis to compare the tweets of younger and older workers in the construction and nursing industries with a view to understanding the common and unique work health and safety challenges they face and subsequent risks to their mental health. A robust observation across each type of analysis was that younger nurses were facing very challenging working conditions (eg, understaffing, high workload, and poor management and communication) that presented barriers to their mental health and well-being. Older nurses faced similar challenges but tended to place greater focus on promoting well-being in their tweets than their younger counterparts. Overall, nurses were unquestionably negatively impacted by the COVID-19 pandemic. In contrast, construction workers showed minimal impacts due to the COVID-19 pandemic. Older construction workers tended to post more negative tweets; however, construction workers in general tended to post less about work-related topics and more about life-related topics, indicating a better work-life balance than was observed in nurses. Younger construction workers posted mostly about life events, suggesting that they faced fewer mental health risks than the other groups.

### Comparison With Prior Work

The most concerning observation that was borne out in each of our different social media analyses was that nurses (and especially younger nurses) were under more work pressure and stress and experienced greater and more intense negative emotions. Moreover, all these emotions were exacerbated by the COVID-19 pandemic. This puts younger nurses at increased risk of burnout and mental harm that may lead to attrition. This pattern of findings is consistent with a recent report that found a 131.5% increase in job vacancies under “health care and social assistance” industries between February 2020 and May 2022 [41]. Although there is a high attrition rate among our older nursing population through retirement, there may be higher attrition among the next generation of nurses through burnout. Previous research has shown that nurses’ mental state plays a critical role in how they respond to situations, patients, and colleagues, as well as their clinical judgment and communication, potentially affecting patient safety [42].

Furthermore, we observed more negative sentiment in tweets about health and well-being topics. This does not align with prior reports that tweets with health care hashtags expressed more positivity and more action-oriented language than non–health care–initiated hashtags [19,43]. It has been suggested that nurses may be reluctant to share negative events on social media due to societal expectations of professionalism from medical experts [13]. It is possible that the discrepancy between prior findings and our results may reflect Australian cultural norms and the extreme pressure that Australian health care workers were experiencing during the study period [13]. Notably, an analysis of tweets comparing UK and US health care workers revealed that while they experienced common challenges during the COVID-19 pandemic, nurses’ experiences in the 2 countries reflected local sociopolitical trends and cultural norms regarding emotional display [19]. Future work should systematically investigate such processes in the Australian context.

Similar to their younger colleagues, older nurses faced challenging work conditions. Furthermore, although they showed signs of elevated distress and negative effects, we found evidence that older nurses possess more effective tools and strategies for dealing with periods of increased uncertainty, stress, and accelerated work demands (eg, emotional regulation and big picture thinking). A common assumption is that older workers are frail and vulnerable; however, evidence suggests that this is an inaccurate stereotype. A lifetime of experience seems to have equipped older workers with resilience and the ability to take a long-term view that permits them to better accommodate periods of instability. These suggestions in our data are consistent with broader research on cognitive aging, which suggests that older adults are skilled at emotion regulation [25]. Moreover, the findings are consistent with prior work on the effects of the COVID-19 pandemic on health care workers, which suggested that younger workers were most at risk of mental illness [26].

Overall, construction workers were less affected by the COVID-19 pandemic. Younger construction workers were mostly focused on posting about life events, including socializing, participating in hobbies, and engaging in recreational activities. Their tweets suggested that they had the best work-life balance, were not preoccupied with work, and showed minimal signs of mental distress. We did find evidence of concern with physical pains and injuries, which is notable given their young age. Older construction workers were more likely to post with more negative sentiment. However, these tweets tended to be regarding issues and events outside of their work, such as politics and current events, rather than about issues concerning their job role or working conditions (in contrast to what was observed for the nurses).

Unlike prior reports [44-46], we did not find evidence of widespread ageism (or reverse ageism) in either industry, although aspects of workplace culture could be improved. Instances of bullying were observed in both industries, originating from various sources, but they did not seem to be widespread. Differences in well-being were primarily related to age, job role, and job type. The findings of this study are compatible with the abovementioned observations and complement the data to provide a detailed insight into the mental health challenges faced by workers in each industry.

This work introduced several innovations. Our team applied social media–based big data analysis to inform our understanding of work health and safety issues. Furthermore, this was the first study to pay particular attention to issues concerning older workers’ mental health by comparing findings and trends across 2 industries as well as comparing patterns in Australia versus overseas.

### Implications

Computational linguistic tools and algorithms are able to reliably predict risks of future mental illness [47,48]. The patterns observed in this study strongly suggested that nurses were experiencing stress and negative emotions, and this was more extreme in younger nurses. A well-developed literature has established which linguistic indicators are reliable predictors of mental ill health (eg, first-person pronouns and negative emotion words) [39]. Language analysis technologies represent an important advancement in mental health care for the prevention and early diagnosis of mental health problems. These tools may aid professionals in identifying at-risk individuals and the follow-up and prognosis of patients [49].

### Limitations

There were several limitations inherent to social media analysis when attempting to extrapolate to real-world work settings. First, not everyone (especially older workers) uses social media; therefore, we cannot assume that the comments, word use patterns, or sentiments of social media users accurately capture those of all construction workers or nurses. To address this limitation, we advocate not relying on any individual tool or method when attempting to develop a deep and holistic understanding of the issues facing an industry or workforce. It is for this reason that, in the present line of research, our team has conducted multiple literature reviews, a survey study, and now a social media analysis before attempting to design an intervention to improve the mental well-being of workers in these 2 industries.

A second limitation is that people whose data were included in this study tend to be those either who have sufficient free time or those who have strong opinions on the subject being studied. This limitation is somewhat circumvented by conducting a big data analysis on tweets that were already publicly available and freely expressed in different contexts. Furthermore, the fact that our observations converge with what we observed in our prior work gives us a high level of confidence that the conclusions drawn from these data are valid.

### Conclusions

This study has advanced our understanding of the well-being of younger and older construction workers and nurses in Australia. The analyses have revealed that nurses (both younger and older) more often communicated about burnout, stress, and ill effects due to poor working conditions during the COVID-19 pandemic than construction workers. However, older nurses tended to promote well-being in their tweets more than their younger counterparts, suggesting that they could be more resilient and better able to adapt and accommodate periods of instability. Older construction workers had more negative sentiments than younger workers, who were more focused on communicating about social and recreational activities rather than work matters. These findings will inform the development of interventions that will protect the mental health of workers in highly demanding work environments such as nursing and construction. In a recent strategic report, the WHO [50] advised that social media accounts (eg, Twitter) could be used for monitoring and provisioning training and educational materials for health care professionals and the evaluation of action plans. Our findings support the value of analyzing social media posts to gain insights into the well-being of workers in target industries and during key social challenges. These tools, and the evidence they generate, should be used to improve the lives of workers, especially those in critical industries.

## Appendix

Multimedia Appendix 1 Summary of users and tweets collected during the study period (January 2018 to June 2022).

Multimedia Appendix 2 Summary of the hashtag topics used by younger and older workers in the nursing and construction industries.

Multimedia Appendix 3 Distribution of the top 200 hashtag topics used by nurses (top panel) and construction workers (bottom panel) expressed as a percentage of the 10 most popular categories.

Multimedia Appendix 4 Average (SE) number of likes, replies, and retweets for tweets with hashtag topics related to health and well-being, with other hashtag topics and topics without hashtags.

Multimedia Appendix 5 Summary of the monogram keywords used in tweets by younger and older nurses and construction workers.

Multimedia Appendix 6 Average (SE) number of likes, replies, and retweets for tweets with and without health and well-being keywords.

Multimedia Appendix 7 Word clouds summarizing the monogram keywords related to physical and mental health issues and well-being for younger nurses (top left), older nurses (top right), younger construction workers (bottom left), and older construction workers (bottom right).

Multimedia Appendix 8 Summary of the bigram keywords used by younger and older nurses and construction workers.

Multimedia Appendix 9 Word clouds summarizing the bigram keywords related to physical and mental health issues and well-being for younger nurses (top left), older nurses (top right), younger construction workers (bottom left), and older construction workers (bottom right).

Multimedia Appendix 10 Descriptive statistics of the Linguistic Inquiry and Word Count scores for the basic summary variables: analytical thinking, clout, authenticity, and emotional tone. Average scores and SE are calculated based on the text from each tweet.

Multimedia Appendix 11 Percentage of words falling within the Linguistic Inquiry and Word Count categories: first-person singular pronouns, drives, cognition, affect, and social. Average percentage and SE are calculated based on the text from each tweet.

Multimedia Appendix 12 Percentage of words falling within the Linguistic Inquiry and Word Count categories: positive emotional tone, negative emotional tone, positive emotion, negative emotion, health, fatigue, illness, wellness, and mental. Average percentage and SE are calculated based on the text from each tweet.

Multimedia Appendix 13 Percentage of words falling within the Linguistic Inquiry and Word Count categories: health, fatigue, illness, wellness, and mental. Average percentage and SE are calculated based on the text from each tweet containing health and well-being keywords. Error bars show SE values.

Multimedia Appendix 14 Percentage of tweets with negative, neutral, and positive sentiments for younger and older nurses and construction workers, grouped by with and without keywords related to health and well-being in the tweets.

Multimedia Appendix 15 Percentage of words falling within the Linguistic Inquiry and Word Count categories: health, fatigue, illness, wellness, and mental. Average percentage and SE are calculated based on the text from each tweet containing health and well-being keywords.

## Abbreviations

LIWC: Linguistic Inquiry and Word Count
WHO: World Health Organization

## Data Availability

The data sets generated and analyzed during this study are available in the Western Sydney University Research Direct repository [51].
