# Supplementary material for: Investigating Health and Well-Being Challenges Faced by an Aging Workforce in the Construction and Nursing Industries: Computational Linguistic Analysis of Twitter Data
Source: J Med Internet Res. 2024 Jun 5;26:e49450. doi: 10.2196/49450 (PMC11187510; doi:10.2196/49450)
Supplement: Multimedia Appendix 1 [file jmir_v26i1e49450_app1.docx]

Summary of collected users and tweets during the study period (January 2018 – June 2022).

| Category |  | Nursing |  | Construction |  |
| --- | --- | --- | --- | --- | --- |
|  |  | User | Tweets | User | Tweets |
|  |  |  |  |  |  |
| Younger | Australia | 68 | 157,532 | 68 | 105,448 |
|  | Worldwide | 69 | 520,825 | 68 | 303,463 |
| Older | Australia | 28 | 65,732 | 24 | 40,001 |
|  | Worldwide | 35 | 180,898 | 35 | 131,739 |
| Total |  | 200 | 924,987 | 195 | 580,651 |
