# Supplementary material for: Investigating Health and Well-Being Challenges Faced by an Aging Workforce in the Construction and Nursing Industries: Computational Linguistic Analysis of Twitter Data
Source: J Med Internet Res. 2024 Jun 5;26:e49450. doi: 10.2196/49450 (PMC11187510; doi:10.2196/49450)
Supplement: Multimedia Appendix 2 [file jmir_v26i1e49450_app2.docx]

Summary of hashtag topics used by younger and older workers in nursing and construction.

| Category | Nursing |  | Construction |  |
| --- | --- | --- | --- | --- |
|  | Unique hashtag topics | Total hashtag topics | Unique hashtag topics | Total hashtag topics |
|  |  |  |  |  |
| Younger | 35,402 | 152,570 | 23,605 | 23,605 |
| Older | 13,897 | 64,940 | 77,929 | 77,929 |
| Total | 45,480 | 217,510 | 11,126 | 11,126 |
