# Supplementary figures and images for: Investigating Health and Well-Being Challenges Faced by an Aging Workforce in the Construction and Nursing Industries: Computational Linguistic Analysis of Twitter Data
Source: J Med Internet Res. 2024 Jun 5;26:e49450. doi: 10.2196/49450 (PMC11187510; doi:10.2196/49450)

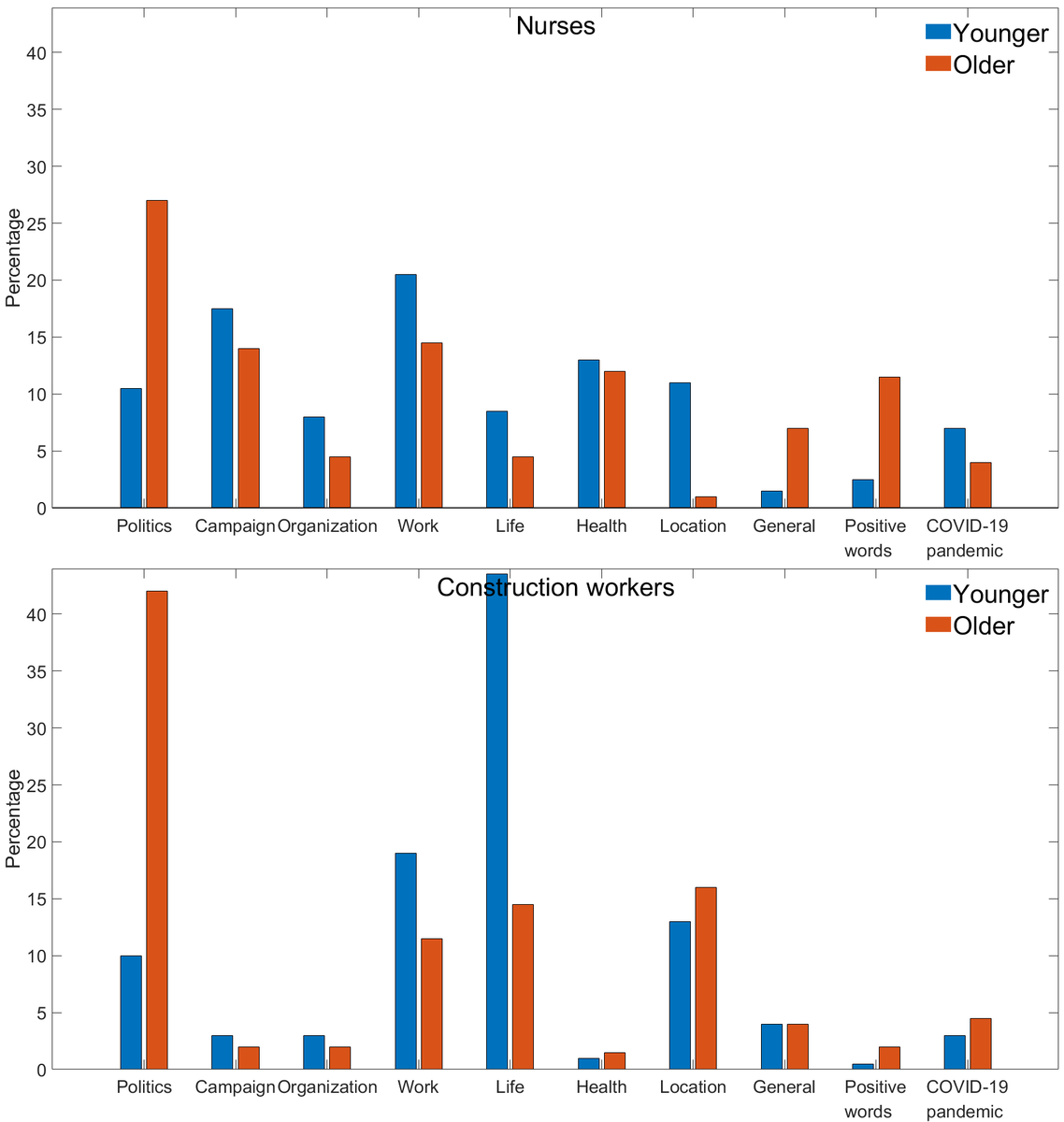

Supplement: Multimedia Appendix 3 [file jmir_v26i1e49450_app3.png]

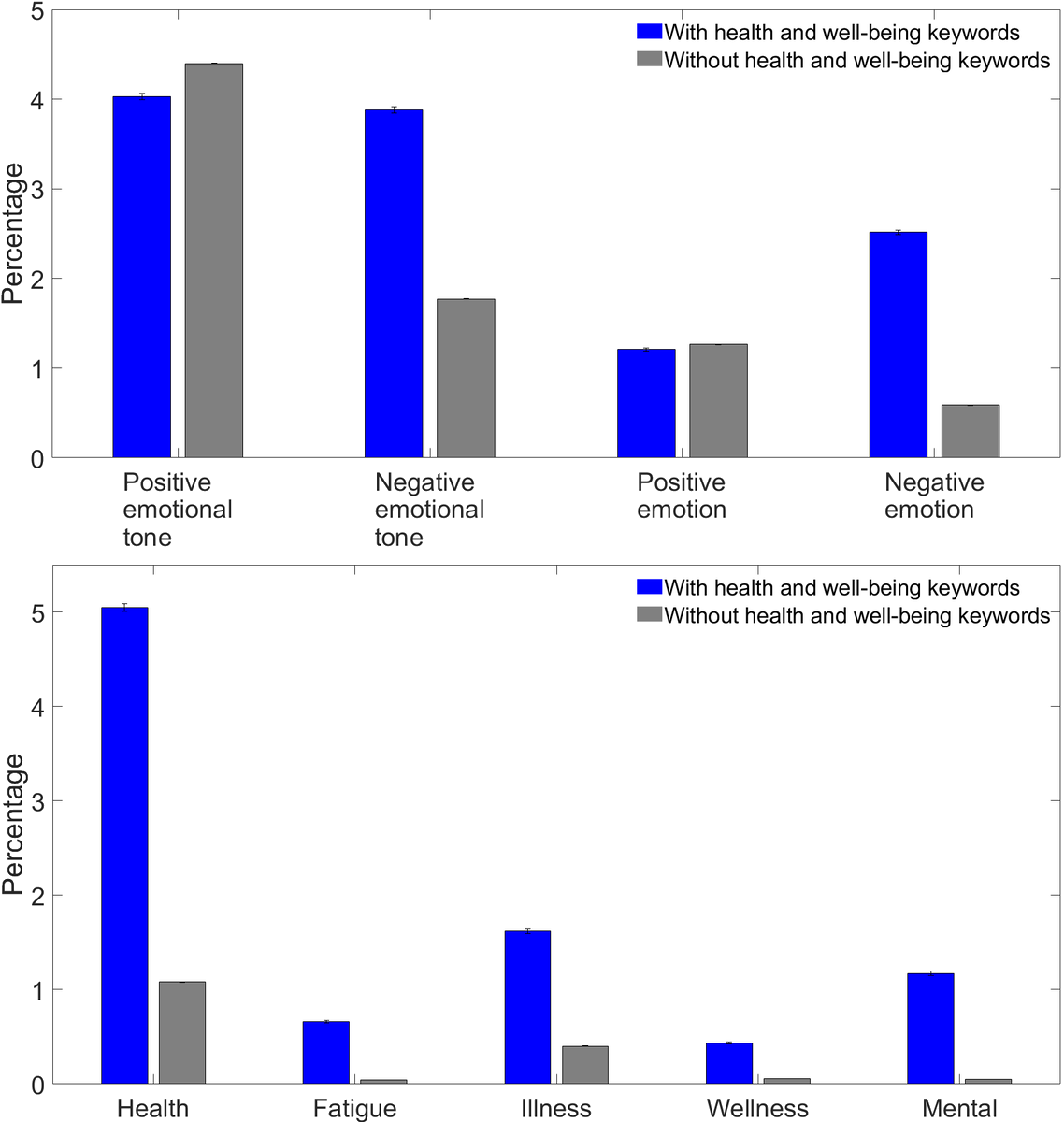

Supplement: Multimedia Appendix 12 [file jmir_v26i1e49450_app12.png]

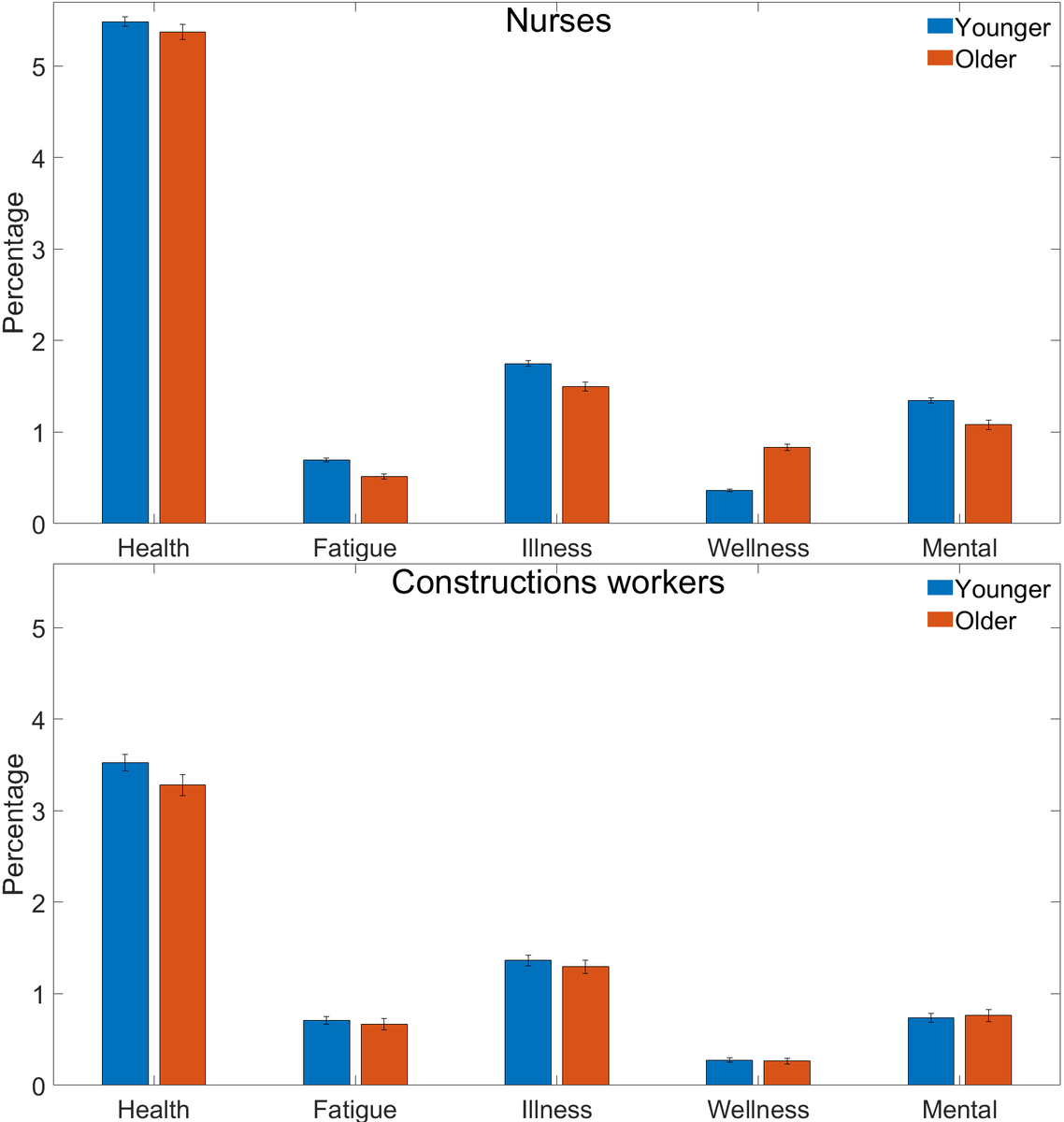

Supplement: Multimedia Appendix 13 [file jmir_v26i1e49450_app13.png]

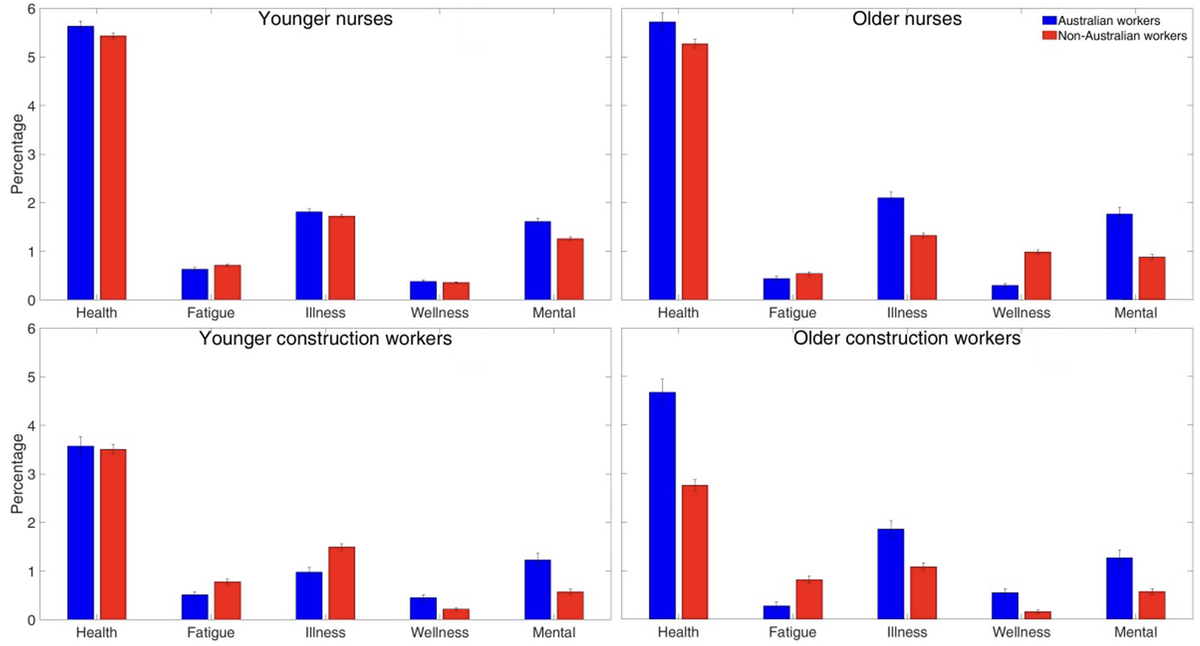

Supplement: Multimedia Appendix 15 [file jmir_v26i1e49450_app15.png]
