# Supplementary material for: Investigating Health and Well-Being Challenges Faced by an Aging Workforce in the Construction and Nursing Industries: Computational Linguistic Analysis of Twitter Data
Source: J Med Internet Res. 2024 Jun 5;26:e49450. doi: 10.2196/49450 (PMC11187510; doi:10.2196/49450)
Supplement: Multimedia Appendix 4 [file jmir_v26i1e49450_app4.docx]

Averaged (and stand error) number of likes, replies and retweets for tweets with hashtag topics related to health and wellbeing, with other hashtag topics and without hashtag topics.

|  | Tweets with health and wellbeing hashtag topics | Tweets with other hashtag topics | Tweets without hashtag topics |
| --- | --- | --- | --- |
|  |  |  |  |
| Likes | 18.3 (3.5) | 13.8 (0.4) | 9.2 (0.3) |
| Replies | 1.03 (0.09) | 1.16 (0.21) | 0.87 (0.01) |
| Retweets | 3.19 (0.57) | 2.90 (0.09) | 1.34 (0.006) |
