# Supplementary material for: Investigating Health and Well-Being Challenges Faced by an Aging Workforce in the Construction and Nursing Industries: Computational Linguistic Analysis of Twitter Data
Source: J Med Internet Res. 2024 Jun 5;26:e49450. doi: 10.2196/49450 (PMC11187510; doi:10.2196/49450)
Supplement: Multimedia Appendix 5 [file jmir_v26i1e49450_app5.docx]

Summary of monogram keywords in tweets by younger and older nurses and construction workers.

|  | Nursing |  | Construction |  |
| --- | --- | --- | --- | --- |
|  | Number of words | Number of words after removing stop words | Number of words | Number of words after removing stop words |
|  |  |  |  |  |
| Younger | 10,731,158 | 3,979,076 | 5,228,673 | 2,167,081 |
| Older | 4,305,999 | 1,642,850 | 3,562,596 | 1,287,634 |
| Total | 15,037,157 | 5,621,926 | 8,791,269 | 3,454,715 |
