# Supplementary material for: Investigating Health and Well-Being Challenges Faced by an Aging Workforce in the Construction and Nursing Industries: Computational Linguistic Analysis of Twitter Data
Source: J Med Internet Res. 2024 Jun 5;26:e49450. doi: 10.2196/49450 (PMC11187510; doi:10.2196/49450)
Supplement: Multimedia Appendix 6 [file jmir_v26i1e49450_app6.docx]

Averaged (and stand error) number of likes, replies and retweets for tweets with and without health and wellbeing keywords.

| n | Tweets with health and wellbeing keywords | Tweets without health and wellbeing keywords |
| --- | --- | --- |
|  |  |  |
| Likes | 22.2 (3.2) | 9.6 (0.3) |
| Replies | 1.27 (0.09) | 0.90 (0.01) |
| Retweets | 3.55 (0.77) | 1.51 (0.06) |
