# Supplementary material for: Investigating Health and Well-Being Challenges Faced by an Aging Workforce in the Construction and Nursing Industries: Computational Linguistic Analysis of Twitter Data
Source: J Med Internet Res. 2024 Jun 5;26:e49450. doi: 10.2196/49450 (PMC11187510; doi:10.2196/49450)
Supplement: Multimedia Appendix 7 [file jmir_v26i1e49450_app7.docx]

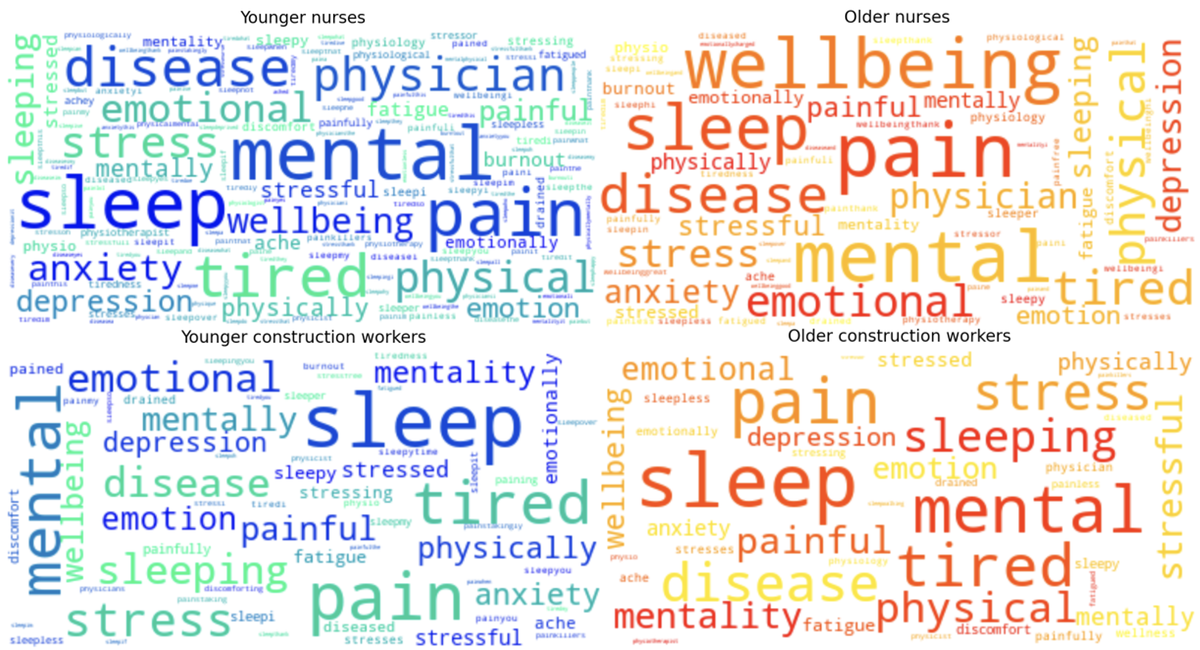


Word clouds summarising monogram keywords related to physical and mental health issues and wellbeing for younger nurses (top left), older nurses (top right), younger construction workers (bottom left) and older construction workers (bottom right).
