# Supplementary material for: Investigating Health and Well-Being Challenges Faced by an Aging Workforce in the Construction and Nursing Industries: Computational Linguistic Analysis of Twitter Data
Source: J Med Internet Res. 2024 Jun 5;26:e49450. doi: 10.2196/49450 (PMC11187510; doi:10.2196/49450)
Supplement: Multimedia Appendix 8 [file jmir_v26i1e49450_app8.docx]

Summary of bigram keywords used by younger and older nurses and construction workers.

|  | Nursing |  | Construction |  |
| --- | --- | --- | --- | --- |
|  | Number of words | Number of words after removing stop words | Number of words | Number of words after removing stop words |
|  |  |  |  |  |
| Younger | 12,675,764 | 1,490,264 | 6,081,238 | 891,042 |
| Older | 4,994,538 | 665,006 | 3,932,419 | 481,886 |
| Total | 17,670,302 | 2,155,270 | 10,013,657 | 1,372,928 |
