# Supplementary material for: Investigating Health and Well-Being Challenges Faced by an Aging Workforce in the Construction and Nursing Industries: Computational Linguistic Analysis of Twitter Data
Source: J Med Internet Res. 2024 Jun 5;26:e49450. doi: 10.2196/49450 (PMC11187510; doi:10.2196/49450)
Supplement: Multimedia Appendix 10 [file jmir_v26i1e49450_app10.docx]

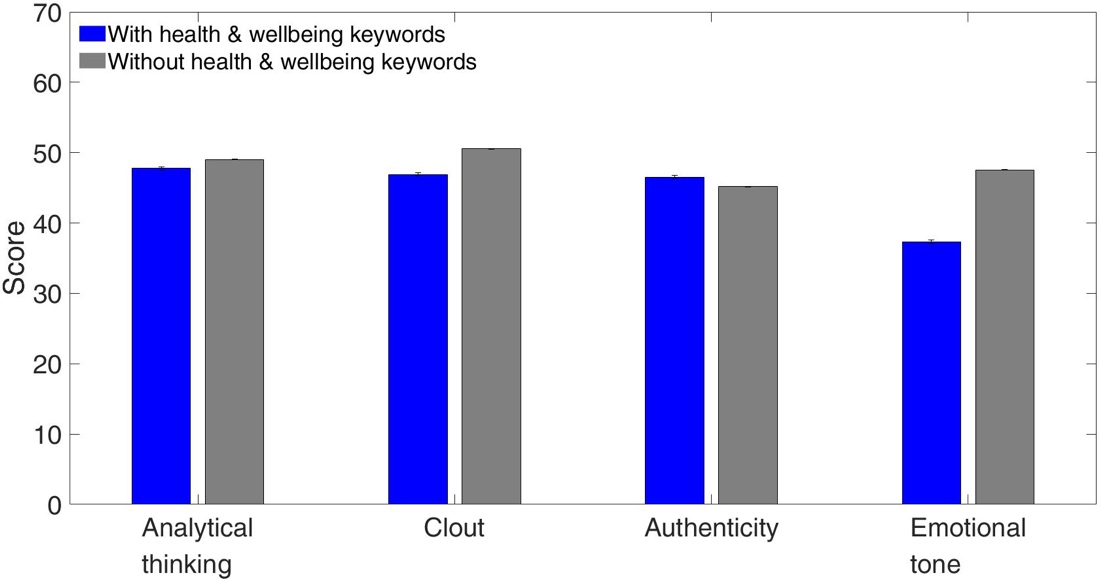


Descriptive statistics of Linguistic Inquiry and Word Count scores for the basic summary variables: analytical thinking, clout, authenticity and emotional tone. Average scores and standard error are calculated based on the text from each tweet.
