# Supplementary material for: Investigating Health and Well-Being Challenges Faced by an Aging Workforce in the Construction and Nursing Industries: Computational Linguistic Analysis of Twitter Data
Source: J Med Internet Res. 2024 Jun 5;26:e49450. doi: 10.2196/49450 (PMC11187510; doi:10.2196/49450)
Supplement: Multimedia Appendix 14 [file jmir_v26i1e49450_app14.docx]

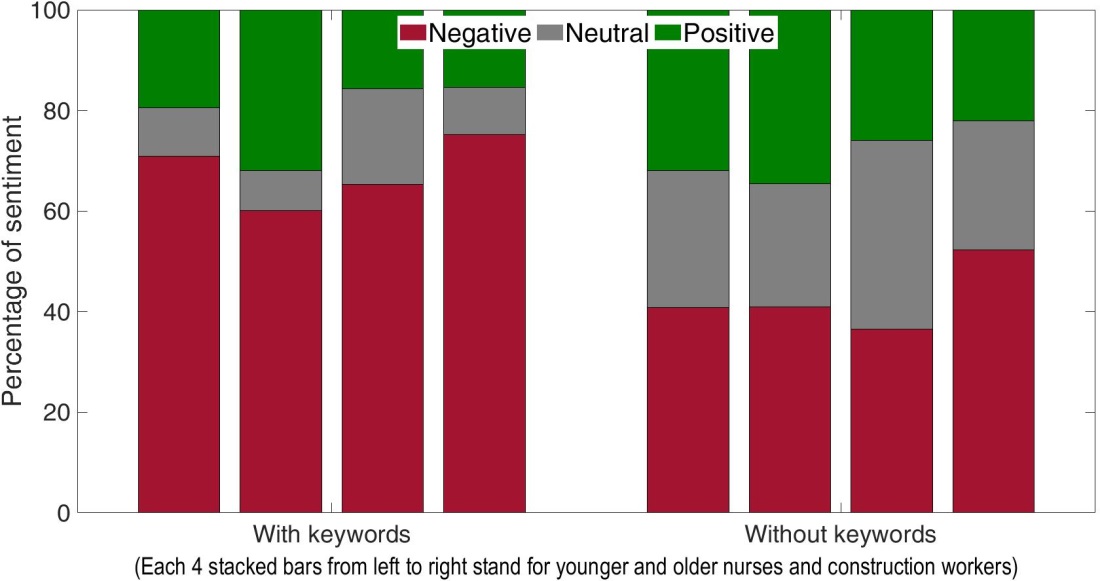


Percentage of tweets with negative, neutral and positive sentiment for younger and older nurses and construction workers, grouped by with and without health and wellbeing related keywords in the tweets.
